# Profiles of sedentary and non-sedentary young men – a population-based MOPO study

**DOI:** 10.1186/s12889-015-2495-6

**Published:** 2015-11-23

**Authors:** Riitta Pyky, Anna-Maiju Jauho, Riikka Ahola, Tiina M. Ikäheimo, Heli Koivumaa-Honkanen, Matti Mäntysaari, Timo Jämsä, Raija Korpelainen

**Affiliations:** Department of Sports and Exercise Medicine, Oulu Deaconess Institute, Albertinkatu 18A, P. O. Box 365, 90100 Oulu, Finland; Center for Life Course Health Research, Faculty of Medicine, University of Oulu, Oulu, Finland; Research Unit of Medical Imaging, Physics and Technology, Faculty of Medicine, University of Oulu, Oulu, Finland; Center for Environmental and Respiratory Health Research, University of Oulu, Oulu, Finland; Infotech Oulu, University of Oulu, Oulu, Finland; Medical Research Center Oulu, Oulu University Hospital and University of Oulu, University of Oulu, Oulu, Finland; Department of Psychiatry, Institute of Clinical Medicine, University of Eastern Finland, Kuopio, Finland; Department of Child Psychiatry, Institute of Clinical Medicine, University of Oulu, Oulu, Finland; Departments of Psychiatry, Kuopio University Hospital (KUH), Kuopio, Finland; Departments of Psychiatry, South-Savonia Hospital District, Mikkeli, Finland; Departments of Psychiatry, North Karelia Central Hospital, Joensuu, Finland; Departments of Psychiatry, SOSTERI, Savonlinna, Finland; Departments of Psychiatry, SOTE, Iisalmi, Finland; Departments of Psychiatry, Lapland Hospital District, Rovaniemi, Finland; Centre for Military Medicine, The Finnish Defence Forces, Helsinki, Finland

**Keywords:** Sitting, Male, Disordered eating behavior, Mood, Cluster, Physical activity

## Abstract

**Background:**

Sedentary behavior is associated with poor well-being in youth with adverse trajectories spanning to adulthood. Still, its determinants are poorly known. Our aim was to profile sedentary and non-sedentary young men and to clarify their differences in a population-based setting.

**Methods:**

A total of 616 men (mean age 17.9, SD 0.6) attending compulsory conscription for military service completed a questionnaire on health, health behavior, socioeconomic situation and media use. They underwent a physical (body composition, muscle and aerobic fitness) and medical examination. Profiles were formed by principal component analysis (PCA).

**Results:**

A total of 30.1 % men were sedentary (daily leisure-time sitting ≥5 h) and 28.9 % non-sedentary (sitting ≤2 h). The sedentary men had more body fat, more depressive symptoms, but lower fitness and life satisfaction than non-sedentary men. However, according to PCA, profiles of unhealthy eating, life-dissatisfaction, and gaming were detected both among sedentary and non-sedentary men, as well as high self-rated PA and motives to exercise.

**Conclusion:**

Determinants of sedentary and non-sedentary lifestyles were multiple and partially overlapping. Recognizing individual patterns and underlying factors of the sedentary lifestyle is essential for tailored health promotion and interventions.

## Background

Sedentary behavior has been defined as any waking behavior characterized by an energy expenditure ≤1.5 METs while in a sitting or reclining posture [1]. Excessive sedentary time has been associated with mortality independent of moderate-to-vigorous physical activity (MVPA) [2]. Sedentary behavior has also been linked with adverse health effects such as diabetes, cardiovascular disease and obesity [3]. Even replacing sitting with standing in a work environment may have short-term positive effects on cardio metabolic risk factors [4, 5]. Sedentary behavior defined as television- and video viewing has been associated with body mass index, depression, ethnicity, socioeconomic status, parental education and male gender among adolescents [6]. Despite the known benefits of non-sedentary lifestyle on health, sedentary time in the U.S. has been reported to be 7.7 h per day and adolescents (aged 16–19 years) spend almost 60 % of their waking hours sitting [7]. The increased sedentariness is a global challenge. In most of the western countries less than 40 % of children and youth meet sedentary behavior or screen-time guidelines [8].

The association between gender and sedentary behavior is contradictory. Boys have been reported to be less sedentary and participate more in organized sports than girls [8, 9] but in turn, sedentary time due to television, computer and console games may be problematic issue especially for boys [8, 10, 11]. A generally healthy population of young men may engage in risky behaviors such as physical inactivity and unhealthy diet placing them at risk of health conditions in the future [12]. Because sedentary behavior has been shown to have stability over time and can partially be tracked to adulthood [13], the transition period from adolescence to adulthood may be an important period for monitoring and intervening sedentary behavior patterns.

Various factors are associated with sedentariness, but are not well understood among young adults. Determinants of sedentary behavior among young people are still lacking [14]. In addition, it is not known how sedentary young men differ from their more active counterparts. Thus, our aim was to profile sedentary and non-sedentary young men and to clarify their differences in physical, behavioral, social, and environmental factors. In this study sedentary behavior was defined as any waking behavior in a sitting posture and physical activity considered as overall daily physical activity.

## Methods

This cross-sectional study is based on a comprehensive population-based study (MOPO), which aims to promote PA and prevent social marginalization among young conscription-aged men [15]. The civic duty or military service is compulsory for male citizens in Finland and conscription is organized every year concerning boys the year they turn 18. Conscription-aged men provide a large, population-based representative sample of Finnish young men.

### Study population

All conscription-aged men who attended the conscription for military service in the Oulu area in 2010 (*n* = 997) were invited to the present study. The final number of those who agreed to participate was 616 (61.8 % of the population). The study protocol included a medical examination before the conscription, a questionnaire, and physiological measurements at the conscription.

The study was conducted according to the Declaration of Helsinki of 1964, as revised in 2000, and was approved by the Ethical Committee of Northern Ostrobothnia Hospital District (ETTM123/2009). The subjects had the right to refuse to participate or withdraw from the study without any effects on their future health care or military service. Written informed consent was obtained from all participants.

### Questionnaire

The questionnaire included items related to socioeconomic situation, physical health and fitness, mental health, PA and time spent sitting, other health behaviors, and time spent on the Internet.

Socioeconomic situation was classified as full-time employee/student, part-time employee/student, or unemployed. Participants were asked to rate their fitness compared to coeval as significantly lower, somewhat lower, similar, somewhat higher, or significantly higher. They also rated their health as good, pretty good, moderate, pretty poor, or poor. In addition, ICD-10 diagnoses from the medical examination were recorded.

Depressive symptoms were evaluated with Raitasalo’s modification of the widely used Beck Depression Inventory (BDI), which has shown to have high validity compared to the unmodified BDI [16]. The four-item life satisfaction (LS) scale measured overall well-being (happiness, interest in life, feelings of loneliness and ease of living), and has shown to be closely related to many psychometric scales [17] and to be able to predict several health outcomes among adults [18, 19]. The participants were also asked whether they were able to discuss their problems with friends and family (1-5: never – mostly or always) and how often they spent time with friends (1 -5: almost never - almost every day).

PA was assessed by the following question: “Approximately, how much are you on the move per day (i.e., biking or walking to school or work, on breaks in school, household chores, or in hobbies and leisure time, etc.)?” The response alternatives were <1 h, 1–2 h, and >2 h. Daily leisure sitting time was also assessed with a question: “How much do you approximately sit per day outside school or work (for example, watching TV, reading, spending time on a computer, playing video games, and using Internet use)?” The respondents were categorized according to daily sitting time as sedentary (≥5 h/day), moderate (2.1–4.9 h/day) and non-sedentary group (≤2 h/day). Time spent on the Internet and frequency of playing Internet games were also separately asked.

Restrictions for PA were asked on a 5-point scale (1-5: not at all - very much) modified from Nigg et al. [20]. The restrictive factors were grouped by principal component analysis (PCA) as follows: lack of resources (i.e., sports equipment, sports facilities, appropriate group, exercise guidance, money, or poor public transport); lack of personal factors (i.e., interest, sports skills, appropriate sports type, knowledge of how to exercise, or laziness); lack of time or tiredness; and lack of health (i.e., illness or injury).

The motivational factors for PA were also rated on a 5-point scale (1-5: not at all - very important) [20] and classified into four categories: health promotion (i.e., enhancing health, mood or energy; enjoying the good feeling coming from exercising; relieving stress); fitness improvement (i.e., competing; enhancing muscle mass or physical fitness); social reasons (i.e., creating or maintaining social affairs; exercising because of the request of a family member or a friend; increasing the appreciation among friends); and appearance (i.e., enhancing exterior or sexual attraction; losing weight). Respondents were also asked whether physical education at school ignited a spark to exercise with a 5-point scale (1-5: strongly disagree - strongly agree).

Participants were asked about their current behavior pertaining to smoking and snuffing, and binge alcohol drinking was assessed by the following question: “How often do you drink alcohol six servings or more at once?” The men were asked whether they usually ate breakfast and the frequency of weekly intake of vegetables, fruits, and berries, as well as weekly intake of fast food and sweets. The study participants rated their dietary habits with the Finnish school grade scale from 4 (poor) to 10 (excellent).

Disordered eating behavior was assessed by the two subscales of the Eating Disorder Inventory (EDI) [21]. Both the Drive for thinness subscale as well as the Bulimia subscale consists of seven questions with a 6-point Likert scale. The questions concerned binge eating, compensation behavior, emotional eating, and losing/gaining weight. The point distribution – with the exception of the first question (inverse scoring) – was as follows: never, rarely or sometimes = 0 points; often = 1 point; usually = 2 points; always = 3 points.

### Measurements

All participants went through a medical and physiological examination as part of the conscription process. Information on diseases, injuries, and use of medication were collected. Height (cm) was measured with 0.5 cm accuracy using a wall-mounted measuring tape. Waist circumference was measured to the nearest 1 cm with a plastic tape measure midway between the lowest rib and the iliac crest at the end of a gentle expiration while the study participant was standing legs apart. Body composition (weight with 0.1 kg accuracy, body mass index, percentage body fat) was measured by direct segmental multi-frequency bioelectrical impedance analysis (DSM-BIA) (InBody720, Biospace Co., Ltd., Seoul, Korea). During the measurement the subject was standing without shoes and socks and wearing light indoor clothing. The method has been validated against whole-body dual X-ray absorptiometry (DXA) [22]. BMI was calculated by dividing the weight (in kilograms) by the height squared (in meters).

The physical performance tests included a bilateral hand grip strength and aerobic fitness test. Grip strength was measured with a hand dynamometer (Saehan, SAEHAN Corporation, Korea). During the test, the subject was instructed to stand legs apart, with elbow at 90° angle, and to grip the instrument with maximum strength. The best result of the two attempts for each hand was recorded. The mean grip strength for the right and left hands was used in the analysis. Aerobic fitness was evaluated using a fitness test (Polar Fitness Test™, Polar Electro, Finland), which was conducted while the subject was resting comfortably for 5 min. The Polar Fitness Test™ predicts maximal oxygen uptake (mL∙min^-1^∙kg^-1^) from the resting heart rate, heart rate variability, gender, age, height, body weight, and self-assessed PA [23]. The Polar Fitness Test has been compared with an ergo-spirometry for measuring aerobic fitness with high correlation (0.96) and with high accuracy (mean error 6.5 %) [24]. The physical measures were piloted among preceding conscripts (a year earlier) and the results were congruent.

### Statistical analysis

In the analyses, the sedentary group (leisure time sitting ≥5 h/day) was compared with the non-sedentary group (sitting ≤2 h/day). The statistical significance of the differences between these two groups was determined using cross-tabulation and chi-squared test for the categorical variables and the Student’s *t*-test for the continuous variables. The strength of the association between continuous variables was analyzed using the Pearson’s correlation coefficient. Restrictions and motivations for PA were grouped by PCA with Varimax rotation [25]. Using the Varimax rotation method minimizes the number of variables that have high loadings on each component and, as such, simplifies the interpretation of the components. Components (profiles) for sedentary and non-sedentary groups were also formed by PCA with the same method. The profiles were named by the nature of the variables loaded into each component. Furthermore, two criteria were tested: the Kaiser-Meyer-Olkin Measure of Adequacy (KMO), a measure of sampling adequacy (threshold: KMO >0.60) and Bartlett’s test of sphericity, which is used to test the null hypothesis that the variables in the population correlation matrix are uncorrelated (threshold: *p* < 0.05). Variables (*n* = 30) included in the PCA were chosen from 112 variables correlated with sedentary behavior. Selection was based on the maximal amount of variables that can be included in the analysis taking into account the sample size, and only one variable per phenomenon was included. Variables with factor loadings ≥ 0.4 were used to calculate factor scores for each of the factors. The number of components for both sedentary and non-sedentary groups was determined by eigenvalue > 1.5 and visual examination of the scree plots. Missing data in PCA were replaced with the means of the groups. Statistical significance was set at *p* < 0.05. The data were analyzed with PASW Statistics software (SPSS version 18, SPSS Inc.).

## Results

A total of 616 (61.8 %) out of the 997 young men who participated in conscription filled in the questionnaire, 610 (61.2 %) underwent the physiological measurements, and 595 (59.7 %) the medical examination. The characteristics of the study participants are presented in Tables 1, 2 and 3. Information on daily sitting time was received from 595 participants. Altogether 30.1 % (*n* = 179) were classified as sedentary (sitting ≥ 5 h/day) and 28.9 % (*n* = 172) as non-sedentary (sitting ≤ 2 h/day) with mean hours of daily sitting being 6.3 (SD 1.8; 95 % CI) and 1.7 (SD 0.5; 95 % CI), respectively.

**Table 1:** Characteristics of the study population by the sedentary status. Values are means (SD) unless otherwise stated. Variables with italics were included in PCA

Characteristic	All (*n* = 616)	Sedentary, sitting time ≥ 5 h (*n* = 179)	Non-sedentary, sitting time ≤ 2 h (*n* = 172)	p*
Age (year)	17.9 (0.6)	17.9 (0.6)	17.9 (0.6)	0.272
Sosioeconomic status, N [%]				0.034
Full-time student/employee	579 (94.0)	160 (89.4)	166 (96.5)	
Part-time student/employee	10 (1.6)	5 (2.8)	1 (0.6)	
Unemployed	27 (4.4)	14 (7.8)	5 (2.9)	
Daily sitting time (hours)	3.8 (2.1)	6.3 (1.8)	1.7 (0.5)	<0.001
Physical activity, daily hours > 1 h, N [%]	490 (81.2)	128 (71.5)	150 (87.7)	<0.001
BMI (kg/m^2^)	22.9 (3.9)	23.1 (4.8)	22.9 (3.6)	0.908
Waist circumference (cm)	82.2 (9.8)	82.7 (11.7)	81.3 (8.8)	0.740
Body fat (%)	16.0 (7.7)	18.3 (9.0)	14.7 (6.7)	<0.001
Aerobic fitness (Polar Fitness Test, mL∙ min^-1^° kg^-1^)	53.4 (7.4)	51.3 (6.6)	56.1 (7.6)	<0.001
Grip strength (kg)	49 (9)	47 (8)	52 (9)	<0.001
Self-perceived fitness; similar/better, N [%]	449 (73.6)	97 (55.1)	151 (88.8)	<0.001
Self-perceived health; moderate/good, N [%]	567 (95.9)	157 (90.3)	161 (98.1)	<0.001
Depressive symptoms (RBDI)^a^	1.8 (3.7)	3.0 (5.2)	0.7 (1.8)	<0.001
Life satisfaction^b^	7.6 (2.8)	8.5 (3.3)	6.8 (2.3)	<0.001
Self-esteem^c^	6.5 (4.1)	5.3 (3.9)	8.1 (4.0)	<0.001
Diagnosed mental disorders, N [%]	19 (1.9)	11 (6.1)	2 (1.2)	0.020
EDI^d^: Symptoms of bulimia (cut-off 4p), N [%]	34 (6.3)	15 (9.0)	7 (4.7)	0.132
EDI: Drive for thinness, N [%]	46 (8.4)	17 (10.6)	7 (4.7)	0.054
Postponed or exempted from military service due to medical reasons, N [%]	160 (27.6)	56 (34.6)	35 (21.3)	0.008

*p-values (sedentary vs. non-sedentary group) independent samples *t*-test or crosstabs chi-squared tests. Fisher’s Exact Test was used, if *n* ≤ 5

^a^Modified Beck’s questionnaire (RBDI): higher score indicating more depressive symptoms

^b^Life satisfaction: higher score indicating lower life satisfaction

^c^Self-esteem (RBDI): higher score indicating better self-esteem

^d^Eating Disorder Inventory (EDI)

Numbers do not match due to missing values

**Table 2:** Lifestyle of the study population by the sedentary status. Values are N [%]. Variables with italics were included in PCA

Characteristic	All (*n* = 616)	Sedentary, sitting time ≥ 5 h (*n* = 179)	Non-sedentary, sitting time ≤ 2 h (*n* = 172)	p*
Eating breakfast (yes)	391 (68.8)	101 (59.8)	121 (78.1)	<0.001
Self-rated quality of diet (good/excellent)	300 (53.6)	61 (37.2)	104 (69.8)	<0.001
Weekly intake (3–7 times)				
Vegetable/fruit/berry,	287 (50.2)	75 (44.4)	94 (55.6)	0.003
Fast food	299 (52.1)	34 (19.8)	14 (8.9)	0.005
Sweets intake	70 (12.2)	102 (59.3)	66 (42.3)	0.002
Current smoker	166 (26.8)	46 (25.7)	47 (27.3)	0.941
Current snuffer	83 (14.4)	14 (8.3)	32 (20.4)	0.002
Binge alcohol drinking (≥1/week)	134 (23.3)	55 (32.7)	24 (15.5)	0.002
Internet use (≥2 h/day)	150 (26.7)	149 (86.6)	41 (26.1)	<0.001
Playing Internet games (≥1/week)	343 (60.6)	125 (73.5)	66 (44.3)	<0.001
Spent time with friends(<1/month)	21 (3.6)	11 (6.4)	2 (1.3)	0.021
Possibilities to discuss problems with friends and family (rarely/never)	53 (9.0)	25 (14.5)	9 (5.6)	0.007

*p-values (sedentary vs. non-sedentary groups) from independent samples *t*-test or crosstabs chi-squared tests. Fisher’s Exact Test was used, if *n* ≤ 5

Numbers do not match due to missing values

**Table 3:** Motives and barriers to exercise by the sedentary status. Values are N [%]. Variables with italics were included in PCA

Characteristic	All (*n* = 616)	Sedentary, sitting time ≥ 5 h	Non-sedentary, sitting time ≤ 2 h	p*
		(*n* = 179)	(*n* = 172)	
Lack of other personal factors; restricting				
Lack of interest	406 (66.7)	144 (80.9)	86 (50.0)	<0.001
Lack of sports skills	221 (36.7)	73 (42.0)	48 (27.9)	0.006
Lack of appropriate sports type	235 (38.8)	85 (48.0)	52 (30.6)	0.001
Lack of knowledge	199 (32.9)	79 (44.6)	37 (21.6)	<0.001
Laziness	493 (81.2)	162 (91.0)	116 (67.8)	<0.001
Appearance; motivating				
Enhancing exterior	530 (87.5)	151 (85.8)	155 (91.2)	0.118
Enhancing sexual attraction	487 (81.7)	130 (76.0)	143 (86.7)	0.012
Losing weight	357 (58.5)	106 (59.9)	102 (59.6)	0.964
Health promotion; motivating				
Enhancing health	590 (96.9)	168 (94.9)	168 (98.2)	0.140
Enhancing mood	535 (88.3)	149 (84.2)	154 (91.7)	0.034
Enhancing energy	555 (91.3)	158 (89.3)	160 (94.1)	0.103
Enjoying the good feeling	550 (90.8)	152 (86.4)	161 (94.2)	0.015
Relieving stress	510 (84.2)	138 (78.0)	154 (91.1)	0.001
Fitness improvement; motivating				
Competing	357 (59.0)	80 (44.9)	116 (68.2)	<0.001
Enhancing muscle mass	567 (93.0)	153 (86.4)	164 (95.9)	0.002
Enhancing physical fitness	566 (93.6)	160 (89.4)	159 (94.1)	0.113
Physical education motivated to exercise	115 (19.0)	22 (12.6)	43 (25.3)	0.003

*p-values (sedentary vs. non-sedentary groups) from independent samples *t*-test or crosstabs chi-squared tests. Fisher’s Exact Test was used, if *n* ≤ 5. Numbers do not match due to missing values

The non-sedentary men had significantly less body fat, better aerobic fitness and grip strength, less depressive symptoms, and better life satisfaction (Table 1). Their self-rated health and fitness was better, their eating habits were healthier, and they spent less time on the Internet. Furthermore, they reported less binge drinking (*p* = 0.002) but they used snuff more often (*p* = 0.002) compared with the sedentary men. Eighty-eight percent (88 %) of the non-sedentary group fulfilled the daily recommendation of 60 min of exercise, compared with 72 % (*p* < 0.001) of the sedentary group.

In addition, restrictions for PA in sedentary men included more often personal factors such as lack of sport skills or laziness than in non-sedentary men, but improving fitness motivated the non-sedentary men more frequently (Table 3).

### Results of the PCA solution for sedentary and non-sedentary young men

Different profiles were observed for both sedentary and non-sedentary men (Tables 4 and 5). The profiles for sedentary men were: “exercising but sitting,” “feeling unhappy,” “symptoms of disordered eating,” “being unfit with exterior motivation,” and “gaming.” The profiles for non-sedentary men were “exercising,” “feeling unhappy,” “gaming,” “unhealthy diet,” and “symptoms of disordered eating.” Among the sedentary profile “exercising but sitting”, the amount of sitting was considerable, but PA, fitness and health were perceived as good. Positive experiences of physical education at school were also reported in this profile. Unhealthy habits, such as indications of disordered eating, unhealthy diet, and excessive time spent on the Internet and Internet games were found among both the sedentary and non-sedentary subjects. Life dissatisfaction and lack of support of friends and family were observed independent of the time spent sitting. Motivation to exercise was reported irrespective of sedentary behavior.

**Table 4:** Profiles of sedentary men and factor loadings of the variables based on the principal component analysis

Variable	1	2	3	4	5
	Exercising but sitting	Feeling unhappy	Symptoms of disordered eating	Being unfit with exterior motivation	Gaming
Motivating: Fitness improvement	0.736				
High self-rated PA	0.676				
Not restricting: Lack of personal factors	0.633				
Physical education ignited the spark to exercise	0.601				
Good self-rated fitness	0.527				
Good self-rated health	0.480				
Dissatisfaction with life		0.803			
Low self-esteem		0.686			
Depressive symptoms		0.644			
Cannot discuss problems with friends or family		0.608			
Diagnosed mental disorders		0.493			
Signs of bulimia			0.874		
Drive to be thin			0.833		
Regular snuffing			0.591		
High body fat percentage				0.769	
Low measured aerobic fitness				0.657	
Motivating: Appearance				0.534	
High daily time on the Internet					0.846
Playing a lot of Internet games					0.779
Variance explained (%)	14.764	8.263	6.341	6.258	4.991
Eigenvalues	4.725	2.644	2.029	2.003	1.597

Kaiser-Meyer-Olkin Measure of Sampling Adequacy 0,663

Bartlett’s Test of Sphericity, Sig. 0.000

**Table 5:** Profiles of non-sedentary men and factor loadings of the variables based on the principal component analysis

Variable	1	2	3	4	5
	Exercising	Feeling unhappy	Gaming	Unhealthy diet	Symptoms of disordered eating
Good self-rated fitness	0.818				
High self-rated physical activity	0.756				
Motivating: Fitness improvement	0.681				
Good self-rated health	0.666				
Good measured aerobic fitness	0.661				
Not restricting: Lack of personal factors	0.500				
Good self-rated diet	0.481				
Spending little time with friends		0.795			
Dissatisfaction with life		0.730			
Cannot discuss problems with friends or family		0.677			
Depressive symptoms		0.620			
Low self-esteem		0.565			
High daily time on the Internet			0.781		
Playing a lot of Internet games			0.747		
Not motivating: Health promotion			0.410		
No regular breakfast				0.724	
Low intake of vegetable, berry, or fruit				0.649	
Higher probability to be unemployed				0.476	
Signs of bulimia					0.839
Drive to be thin					0.610
Variance explained (%)	17.056	8.351	5.769	5.644	4.992
Eigenvalues	5.458	2.672	1.846	1.806	1.598

Kaiser-Meyer-Olkin Measure of Sampling Adequacy 0.738; Bartlett’s Test of Sphericity, Sig. 0.000

## Discussion

In this cross-sectional population-based study of young men, PCA was used for the first time to profile sedentary and non-sedentary young men taking into account a variety of physical, behavioral, social, environmental and health factors. It resulted in profiles that differed somewhat by the sedentary status. The physical performance and mental health of sedentary men were worse than in non-sedentary young men. Those with high leisure sitting time were not necessarily physically inactive and both sedentary and non-sedentary men declared motives to exercise.

Sedentary and PA patterns have been studied especially among children and adolescents, but mainly in respect to dietary patterns [26, 27]. Moreover, previous studies have mostly focused on different actions (i.e., TV viewing, reading, computer usage, homework) within sedentary behavior. Our data had more comprehensive perspective, which included leisure-time sitting, physical performance, other health behaviors as well as both physical and mental health. Thus, these variables enabled to identify groups not been studied before, when resulting in five profiles both for sedentary and non-sedentary group.

Formerly, Gorely et al. [27] have detected three different sedentary clusters “TV viewers”, “computer users”, and “homeworkers,” in addition to “semi-active socializers,” and “actives” among male adolescents. They did not identify a sedentary group, which was exercising, as in our study (i.e. “exercising but sitting”). However, Wang et al. [26] identified five groups of students with unique sedentary and PA patterns, differing by age, screen time and homework time. One of the groups had a sedentary pattern (due to playing computer/video games), but, still, with high PA level. Also in another study, adolescents participating in skating as well as in video gaming had the highest odds of meeting the PA recommendations [28]. Thus, time spent on the internet and engagement in internet games can be associated with both sedentary and non-sedentary lifestyles. Previous studies have also suggested that sedentary behavior and PA do not necessarily displace one another and may coexist. For example the meta-analysis of Pearson et al. [29] found only a minor, inverse association between sedentary behavior and PA among young people. In our study, high levels of PA were found both in sedentary and non-sedentary young men. In the group of “exercising but sitting” high PA, good health together with considerable amounts of sitting (i.e. a health risk) were detected. Thus, our results and previous studies [30] may indicate that adolescents might not recognize their low PA level, or they are not aware of or do not care about its adverse health effects.

Also less sedentary behavior may involve other unhealthy habits such as poor dietary behaviors [31]. Indeed, in the present study unhealthy diet or symptoms of disordered eating were found regardless of sedentary status. On the other hand, our non-sedentary profile “unhealthy diet” (without regular breakfast or use of vegetables) included young men with higher probability of being unemployed, while higher PA has been previously related to being employed [32]. This discrepancy might be somewhat due to the lack of objectively measured PA and sitting time in our study.

None of these other studies measured mental health or wellbeing. In our study, in addition to “gaming”, also “feeling unhappy” – parallel to “symptoms of disordered eating” - were found regardless of sedentary status. Since prolonged sitting [33] and life dissatisfaction have been linked to several adverse health outcomes [18, 19, 34], special attention should be paid to those sedentary young men, who are not feeling well (profile “feeling unhappy”). Also their eating behaviors should be evaluated.

It is not surprising that the non-sedentary young men were in better physical condition, were feeling better, and had healthier lifestyles than the sedentary men. Also previously, leisure sitting time (self-report) has been inversely linked with physical fitness (measured) [35], while sedentary time and low PA has been associated with depressive symptoms and mental problems [36, 37]. Physically active adolescents have also been suggested to spend more time with friends [27]. In our study the lack of friends or lack of possibility to discuss with friends was observed regardless of activity level. However, neither healthy diet nor alcohol intake was related to PA in adolescents [38].

When motives to exercise were studied, the sedentary profile “being unfit with exterior motivation” could be the easiest to activate due to their motives being concrete and outcomes (such as losing weight) easy to measure. Previously, body shape and weight management have been inconsistent in motivating especially teenagers and women [39]. In men, competition, social recognition, and fitness improvement motivate to exercise [40], while having fun has motivated young men. Losing weight can motivate physically inactive people, social factors less educated people [41] and improvement of physical performance adults who are already exercising [41]. In our study, non-sedentary men could be activated through fitness improvement but not through health promotion. Lack of personal factors (interest, skills or knowledge) and laziness were a bigger barrier to exercise among sedentary than non-sedentary men. This is in accordance with the study of Ebben et al. [42], who identified laziness and the lack of motivation as barriers to exercise of physically inactive students.

Our results showed that profiles of sedentary young men differ in socioeconomic situation, physical and mental health, health behavior, PA, and restrictions and motives to exercise. Thus, physical motivation of sedentary young men cannot be based solely on the activity level. Individual unhealthy life habits, and barriers and motivations for exercise have to be considered. In our previous study, we also suggested that more individual and tailored activation with appropriate feedback message tactics is needed [43].

The strength of this study was its population-based design including the entire age cohort of young men and extensive data collection (questionnaire/examination/measurements) with high compliance due to obligatory conscription. The measurements (physical performance/body composition) were carried out under controlled conditions. Using PCA, which combines a large amount of variables into profiles, enabled to reveal new associations and new knowledge. It provides also statistical criteria to the use of sub-scales. Still, interpretation of results provided by PCA is partly subjective, which can be considered as a limitation or a strength of the method.

Nevertheless, a limitation was the lack of objectively measured PA and sedentary time and the lack of data on sedentary time in work activities. Questionnaires have shown limited reliability in respect to PA [44], which is generally overestimated [45] and sedentary time underestimated [44]. Information on daily leisure time sitting was not received from 40.3 % of the participants and the proportion of missing data varied from 0 to 56 % between the variables. Daily PA was based on a simple question with 3 response alternatives. Previous studies have, however, suggested that in addition to validity and reliability, the ease of administration is crucial in population-based studies on other health behaviors besides PA [46]. The nature of sitting was not defined, nor the time spent standing measured, even if the latter is associated with improved health [47, 48]. In this study sedentary time was not split into screen-based and non-screen-based sedentary time since the aim was not to investigate which sedentary patterns are harmful or not. Instead, we wanted to evaluate how overall sedentary lifestyle is built up in young men and factors associated with it.

## Conclusions

This study adds to literature on factors underlying sedentary behavior in young men. Sedentary time is a set of multiple behaviors, and on the other hand, non-sedentary lifestyle does not necessarily mean avoiding all possible sedentary actions. In this study we found that there are different profiles in regard to socioeconomic, health, and lifestyle factors among sedentary and non-sedentary young men, even if some factors, i.e. unhealthy habits, gaming, life dissatisfaction, and high self-rated PA, were common and should be recognized both in sedentary and non-sedentary young men. In young men, recognizing individual patterns and underlying factors of sedentary lifestyle and tailoring health promotion for them are essential for effective interventions. These results and profiles can be utilized in planning the future implementation of physical activation of young men. Prospective studies are needed in order to investigate the lifelong trajectories and determinants of sedentary behavior in young men.

## Abbreviations

BDI: Beck Depression Inventory
DSM-BIA: Direct segmental multi-frequency bioelectrical impedance analysis
DXA: Dual X-ray absorptiometry
EDI: Eating Disorder Inventory
KMO: Kaiser-Meyer-Olkin Measure of Adequacy
LS: Life satisfaction
PA: Physical activity
PCA: Principal component analysis
SD: Standard deviation
